# Association of CSF and PET markers of neurodegeneration with electroclinical progression in Lafora disease

**DOI:** 10.3389/fneur.2023.1202971

**Published:** 2023-06-28

**Authors:** Giuseppe d'Orsi, Andrea Farolfi, Lorenzo Muccioli, Orazio Palumbo, Pietro Palumbo, Sergio Modoni, Vincenzo Allegri, Valentina Garibotto, Maria Teresa Di Claudio, Ester Di Muro, Mario Benvenuto, Francesca Bisulli, Massimo Carella

**Affiliations:** ^1^Neurology Unit, Fondazione IRCCS Casa Sollievo della Sofferenza, San Giovanni Rotondo, Italy; ^2^Nuclear Medicine, IRCCS Azienda Ospedaliero-Universitaria di Bologna, Bologna, Italy; ^3^Department of Biomedical and Neuromotor Sciences, University of Bologna, Bologna, Italy; ^4^Division of Medical Genetics, Fondazione IRCCS Casa Sollievo della Sofferenza, San Giovanni Rotondo, Italy; ^5^Nuclear Medicine Department, Policlinico Riuniti, Foggia, Italy; ^6^Diagnostic Department, University Hospitals of Geneva, CIBM Center of Biomedical Imaging and NIMTLab, University of Geneva, Geneva, Switzerland; ^7^IRCCS Istituto delle Scienze Neurologiche di Bologna, Epilepsy Center (Full Member of the European Reference Network EpiCARE), Bologna, Italy

**Keywords:** follow-up, Lafora disease, electro-clinical features, amyloid biomarkers, neurodegenerative biomarkers, progressive myoclonic epilepsy, 18F-FDG PET

## Abstract

**Purpose:**

To evaluate the electro-clinical features in association with laboratory and instrumental correlates of neurodegeneration to detect the progression of Lafora disease (LD).

**Methods:**

We investigated the electro-clinical longitudinal data and CSF Aβ42, p-tau_181_ and t-tauAg, amyloid, and ^18^F-FDG PET of five unrelated LD families.

**Results:**

Three progressive electro-clinical stages were identified. The early phase was characterized by rare, generalized tonic-clonic and focal visual seizures, followed by the occurrence of myoclonus after a period ranging from 2 to 12 months. The intermediate stage, usually occurring 2 years after the onset of epilepsy, is characterized by a worsening of epilepsy and myoclonus associated with progressive dementia and cerebellar signs. Finally, the late stage, evolving after a mean period of 7 ± 1.41 years from the onset of the disease, was characterized by gait ataxia resulting in bedriddenness, severe dementia, daily/pluri-daily myoclonus, drug-resistant epilepsy, clusters of seizures or status epilepticus, and medical complications. Amyloid (CSF Aβ42, amyloid PET) and neurodegenerative (CSF p-tau_181_ and t-tauAg, FDG-PET) biomarkers indicate a pattern of cognitive impairment of the non-Alzheimer's disease type. A total of 80% of the LD patients showed more severe hypometabolism in the second FDG-PET scan compared to the first scan performed in a lower phase; the lateral temporal lobe and the thalamus hypometabolism were associated with the presence of intermediate or late phase.

**Conclusions:**

Three electroclinical and 18F-FDG PET evolutive stages are useful biomarkers for the progression of LD and could help to evaluate the efficacy of new disease-modifying treatments. The combination of traditional CSF biomarkers improves the diagnostic accuracy of cognitive decline in LD patients, indicating a cognitive impairment of the non-Alzheimer's disease type.

## Key points

- Three evolutive electroclinical stages, including four main progressive symptoms, have been outlined during the natural history of Lafora disease.
- Aβ (CSF Aβ42, amyloid PET) and neurodegenerative (CSF p-tau_181_ and t-tauAg, 18F-FDG PET) biomarkers suggest a pattern of cognitive impairment of non-Alzheimer's disease type.
- 18F-FDG PET is a promising biomarker to track progressive neurodegenerative changes occurring in LD and suggests the possible implication of thalamus in LD pathogenetic mechanisms.

## Introduction

Lafora Disease (LD, OMIM# 254780) is an autosomal recessive progressive myoclonic epilepsy (PME) caused by loss-of-function mutations in either the *EPM2A* (OMIM # 607566) gene, encoding the laforin glycogen phosphatase, or the *NHLRC1* (OMIM # 608072) gene, encoding the malin ubiquitin E3 ligase (1). The absence of either of the two proteins results in a lack of protein degradation targeting the glycogen, thus leading to the altered biosynthesis of the glycogen, which accumulates as inclusions (Lafora bodies) in the neurons, which is arguably responsible for neurodegeneration and disease progression (2). Clinically, LD is a fairly homogenous disease with onset in adolescence in otherwise neurologically normal individuals with tonic-clonic, myoclonic, and focal visual seizures (1, 3). As the disease progresses, LD patients develop myoclonus, ataxia, and rapidly progressive dementia. Death commonly results from status epilepticus, aspiration pneumonia, and other complications of chronic neurodegeneration within a decade after the first symptoms in half of the cases (1, 4, 5).

However, comprehensive data on clinical features and long-term follow-up are scarce due to rapid disease progression or delayed diagnosis (1, 3, 4).

In one of the first LD case series, Tassinari et al. (6) described three electroclinical phases: the first phase was clinically characterized by tonic-clonic seizures with EEG features similar to those shown for generalized genetic epilepsy; the second phase, presenting myoclonus and associated with a progressive slowing of the posterior background, diffuse faster and irregular discharges of spike-waves and focal occipital abnormalities; and finally, a terminal phase, characterized by dementia and diffusely slow EEG with superimposed multiple fast spikes. Subsequently, with the advent of genetic analysis, a few studies (7–12) have demonstrated that patients with *NHLRC1* mutations, compared to patients with *EPM2A* mutations, tended to have a slightly milder clinical course, a later onset, and a slower progression. Nevertheless, this genotype-phenotype correlation was not supported by a recent review, which suggests the possible role of specific mutation types and their interactions with other “modifier genes” (4).

Even though a non-specific treatment for LD is available (13), promising new therapeutic strategies are currently being tested in animal models and will hopefully be available soon for clinical trials (14, 15). Therefore, the identification of biomarkers that can enable an early and correct diagnosis of LD and facilitate the monitoring of disease progression is of paramount importance. Nevertheless, data on the use of biomarkers for the onset and progression are scarce (16–18), and the correlation of CSF and metabolic biomarkers with electro-clinical features during the natural history of LD is lacking.

The purpose of our study is to evaluate the electro-clinical longitudinal features of LD in five unrelated Apulian (Southern Italy) families in association with laboratory and instrumental correlates of neurodegeneration, namely CSF Aβ42, p-tau_181_ and t-tauAg, amyloid, and ^18^F-FDG PET, to detect the features allowing an early diagnosis and the monitoring of the disease progression.

## Methods

We collected and analyzed data from five unrelated LD Apulian families, diagnosed by genetic analysis and prospectively monitored at the Neurology Unit of the Fondazione IRCCS Casa Sollievo della Sofferenza, located in San Giovanni Rotondo (FG), Italy.

### Clinical and video-EEG/polygraphic study

All patients were electro-clinically followed up twice per year. Data from each patient were tabulated and included demographic and general information, details of the epilepsy features; the presence of other symptoms, the evolution of epilepsy; and other symptoms. Clinical progression was assessed using a simplified disability scale (11, 12) based on cognitive performance (assessed by Montreal Assessment, MOCA), residual motor function (ataxia), daily living (ADL), and social abilities, ranging from 1 to 4. Moreover, all patients were administered the Wechsler Intelligence Scales (WISC-R: Wechsler Intelligence Scale for Children-Revised) and a brief neuropsychological test battery covering language, category fluency, attention/processing speed, executive function (WMS-R Digit Span; Trail Making Test; WAIS-R Digit Symbol Test), phonemic fluency, verbal, and visuospatial memory. Myoclonus severity was scored using a simplified myoclonus rating scale (19). The parameters of the video-EEG/polygraphic recordings included video-EEG (electrodes were placed based on the 10-20 International System with bipolar montage); an electromyogram (EMG) of both deltoid muscles, the right and left flexor and extensor muscles of the hand, and both anterior tibialis muscles; an EKG; and thoracic respiration (monitored using a strain gauge). Signals were acquired digitally (sampling frequency: 512 Hz; band-pass filters: 1.6–210 Hz; Nihon Kohden, Tokyo, Japan). The relationship between EEG and EMG bursts (myoclonus) was analyzed by applying jerk-locked back-averaging.

### Genetic analysis

Sample genomic DNA extracted from peripheral blood was analyzed by Next Generation Sequencing (NGS) as described in a previous study (3). Nucleotide variants identified as pathogenetic were reported in the Leiden Open Variation Databases (LOVD) (https://databases.lovd.nl/shared/variants/0000665920#00007173, https://databases.lovd.nl/shared/individuals/00303262, https://databases.lovd.nl/shared/variants/0000667827#00000102).

### Cerebrospinal fluid (CSF) analysis

CSF was collected in 12 ml polypropylene tubes and centrifuged at 2,171 g for 10 min (3,400 rpm) within 2 h. CSF was aliquoted in polypropylene tubes and temporarily stored at −22°C to quantify Aβ_1 − 42_, p-tau_181_, and Tau concentrations within 1 month after collection (Innotest ELISA; Innogenetics, Ghent, Belgium). The ROC curves were used to determine the cutoffs for every single value, and a logarithm that correlates the IATI index ß-amyloid 1–42/(240 + 1.18 x t-tau) with the value of p-tau_181_ was calculated.

### Magnetic resonance imaging (MRI)

All LD patients underwent brain MRI (1.5-3 Tesla System).

### PET study

Brain FDG-PET imaging was performed using a dedicated PET/CT 3D system. In compliance with the European guidelines (20), ^18^F-FDG was administered at a dose of 125–250 MBq, with image acquisition 45–60 min after injection. The images were corrected for scattering and attenuation and rebuilt using an OSEM 3D algorithm.

In patients 1, 2, 3, and 4, FDG-PET was repeated during disease progression, 12–18 months after the first FDG-PET examination.

#### SPM image analysis

FDG-PET images were preprocessed using the Statistical Parametric Mapping 12 (SPM12) software (Wellcome Department of Imaging Neuroscience, Institute of Neurology, London, UK) running in Matlab 9.11.0 (Natick, Massachusetts: The MathWorks Inc.). A reference control group made up of 154 subjects (86 female and 69 male subjects) with a median age of 63 years (age range: 12–84 years) was considered (database AIMN-NSG). Images were placed in the standard Montreal Neurological Institute (MNI) space using an FDG-PET dementia-specific template for spatial normalization based on images derived from both neurological patients and age-matched controls (https://www.fil.ion.ucl.ac.uk/spm/ext/). Each scan was tested for relative hypometabolism by comparison with the reference group on a voxel-by-voxel basis using the general linear model through the two-sample *t*-test design of SPM12. The regional differences in glucose metabolism compared to the control group were shown as a t-statistic for each voxel (SPM-t maps). Uncorrected *p*–values of <0.05 were analyzed, with a cluster level above 100 voxels. Significant reductions or increases in metabolism were shown on a yellow-red scale, ranging from preserved metabolism to severe hypometabolism or hypermetabolism. SPM-t colored displays of FDG-PET hypometabolism were superimposed on MRI T1-weighted structural brain scans in the MNI space, with 4 mm slices on the z-axis.

#### Visual image interpretation

Two nuclear medicine physicians with at least 5 years of experience in neuroimaging (A.F. and V.A.) and blinded to clinical data independently evaluated anonymised FDG-PET images, which were axially reoriented parallel to the anterior commissure-posterior commissure line, according to the method described by Friston et al. (21). Using a graduated scale ranging from preserved metabolism to severe hypometabolism, 31 regions were systematically analyzed. Based on both visual interpretation and SPM-t maps, they were asked to make a diagnosis and indicate their degree of diagnostic confidence on a 3-point Likert scale. In cases of disagreement, the case was discussed to reach a consensus for a final interpretation.

### Statistical analysis

The patients' clinical and demographical features were reported as frequencies and percentages or as the mean and standard deviation for categorical and continuous variables. Group comparisons were performed using the Fisher exact test and the *t*-test for categorical and continuous variables. Two-sided *P*-values of <0.05 were considered to be statistically significant. The disease stages were grouped as 1 and 2–3 for the *t*-test and multivariable regression analysis. The Kruskal–Wallis test was used for metabolism during the progressive phases of the disease. All statistical analyses were performed using the computing environment R (R Development Core Team, version 3.3.2).

The local Ethics Committee on Human Experimentation approved the study, and written informed consent was obtained from the patient's relatives.

## Results

Six LD patients (4 male patients and 2 female patients; mean age: 21 ± 4 years, median 22 years, range 14–25 years) from five unrelated Apulian families were investigated. The mean age at the LD onset was 12.5 ± 1 years (median 13, range 10–14), while the mean age at LD diagnosis was 15 ± 2 years (median 16 years, range 13–18 years).

**Figure 1:**
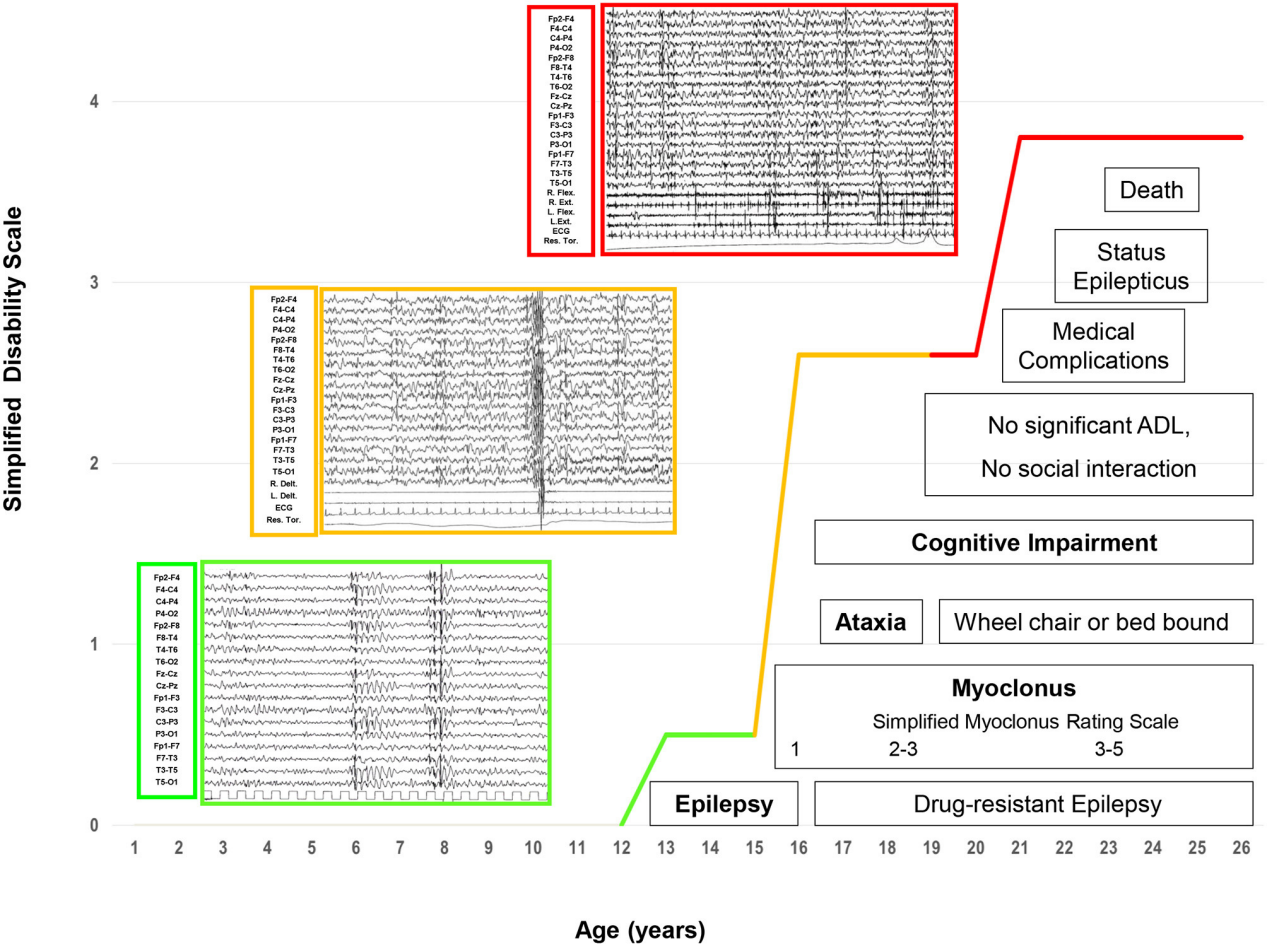
The electroclinical phases of LD patients. Four main and progressive symptoms (epilepsy, myoclonus, ataxia, and dementia) are included in three evolutive electroclinical stages [green line: Phase 1 (early stage); orange line: Phase 2 (intermediate stage); red line: Phase 3 (late stage)].

Three progressive electro-clinical stages (see Figure 1) were identified during the evolutionary course of the disease, and CSF Aβ42, p-tau_181_ and t-tauAg, amyloid PET, and FDG-PET were monitored at the three stages of LD.

Table 1 summarizes the patients' clinical features.

**Table 1:** Clinical features of Lafora disease patients.

**Pt/Sex**	**Genetic features**	**Age early stage onset (y)**	**Age intermediate stage onset (y)**	**Age late stage onset (y)**	**Seizure Type**	**Status epilepticus Type**	**Myoclonus score**	**Dementia**	**Medical complications**	**Disease duration (y)**	**Conditions at last Follow-up**
1/F	EPM2A: c.721C>T p.(Arg241^*^)	13	15	19	Myoclonic, Tonic-Clonic	Myoclonic, Myoclonic-Tonic; NCSE	5	Severe	Dysphagia, aspiration pneumonia, bedsores.	8	Mute and bedridden, with PEG and tracheostomy.
2/M	EPM2A: c.721C>T p.(Arg241^*^)	13	16	22	Myoclonic, Tonic-Clonic	Myoclonic, Myoclonic-Tonic; Focal Motor; Tonic; NCSE	5	Severe	Dysphagia, aspiration pneumonia.	12	Mute and bedridden, with PEG. Death at age 25 from pneumonia.
3/M	EPM2A: c.721C>T p.(Arg241^*^)	10	13	18	Myoclonic, Tonic-Clonic	Myoclonic; Myoclonic-Tonic	5	Severe	Dysphagia, aspiration pneumonia, bedsores.	16	Mute and bedridden, with PEG. Death at age 26 from pneumonia.
4/M	EPM2A: c.243_246del p.(Asp82Argfs^*^7) c.721C>T p.(Arg241^*^)	12	16	–	Myoclonic, Tonic-Clonic	–	4	Moderate	-	5	Moderate ataxia, limited interaction.
5a/F	NHLRC1: c.992del p.(Gly331Glufs^*^3)	13	15	19	Tonic-Clonic	–	5	Severe	Dysphagia, aspiration pneumonia.	11	Mute and bedridden, with PEG. Death at age 24 from SUDEP.
5b/M	NHLRC1: c.992del p.(Gly331Glufs^*^3)	14	17	20	Myoclonic Tonic-Clonic	Tonic-Clonic	4	Moderate	Dysphagia, aspiration pneumonia.	6	Moderate ataxia, limited interaction. Death at age 20 from sepsis.

LD, Lafora disease; y, years; F, female; M, male; PEG, percutaneous endoscopic gastrostomy; NCSE, Non-convulsive SE with coma.

### Electroclinical findings

#### Phase 1: early stage

The electro-clinical presenting symptoms were extensively reported in a previous study (3). Briefly, generalized tonic-clonic seizures, focal visual seizures, and bilateral tonic-clonic seizures had an early onset, and the average elapsed time between the onset of epilepsy and that of other symptoms was 15 months (range 3–24). Epileptic seizures at onset were sporadic and responsive to anti-seizure medication (ASM) monotherapy. Subsequently, after a period ranging from a few weeks to 12 months, patients presented monthly with myoclonic jerks occurring, often but not always, upon awakening. Myoclonic jerks were more marked in the upper body and were more often postural; they became evident as the patients started to move. The mean myoclonus severity score was 1 (ranging from 1 to 2). At the time of the first EEG (at the onset of epilepsy), the background activity was mild and diffusely slow, and sporadic diffuse spike-and-wave (SW) or polyspike-and-wave (PSW) abnormalities, usually with a maximum discharge over the occipital region, became particularly evident during drowsiness and sleep (NREM stages 1–2). Sporadic focal occipital epileptiform abnormalities were also detected in all patients. At the time of the second or third EEG (myoclonus onset), the EEG background activity was slow; diffuse SW, and, more often, PSW bursts, sometimes associated with brief sequences of myoclonic jerks, and occipital SW abnormalities were observed during wakefulness and sleep (NREM stages 1–2); physiological sleep patterns were reduced. The mean disease stage was 0.5 ± 0.5 points (range 0–1).

#### Phase 2: intermediate stage

After 2 years from the onset of epilepsy, this phase was characterized by a worsening of epilepsy and myoclonus associated with a gradual onset of dementia and cerebellar signs. All patients presented daily with myoclonic multi-focal jerks precipitated by movements and, if massive, associated with falls. The mean myoclonus severity score was 3.4 (range 3–4). Tonic-clonic seizures occurred weekly/pluri-monthly, while patients 3, 5a, and 5b also experienced focal visual seizures monthly/pluri-monthly. All patients manifested drug-resistant epilepsy, despite a combination of ASMs and treatment with add-on metformin (13). The EEG background activity slowed further, with intermixed, diffuse, and faster discharges of SW/PSW, during awakening and disorganized sleep. Focal occipital spikes were especially observed in patients 1, 2, and 4 and were accentuated by sleep. Brief sequences of myoclonic jerks were associated with EEG diffuse paroxysms in all patients; in particular, polygraphic recordings revealed action myoclonus and, during posture maintenance, erratic myoclonic jerks at rest. Unilateral myoclonia also occurred without evident EEG correlates; jerk-locked back-averaging EEG analysis triggered by myoclonic jerks revealed a contralateral spike at the centroparietal electrodes in some myoclonia. In cases 3 and 4, intermittent photic stimulation (IPS) triggered bursts of diffuse SW or PSW, which were associated with myoclonic jerks, particularly in the upper limbs, at a 1:1 ratio at frequencies of up to 12–18 Hz. In Patient 1, IPS revealed focal photic reflex myoclonus, and a back-average of the EEG, triggered from the onset of myoclonus by the right extensor muscle of the hand, showed a contralateral positive-negative transient in the frontocentral region that preceded the myoclonus EMG discharge by 18–20 ms.

The average elapsed time between dementia and the onset of epilepsy was 2.4 years (range 1–5); the mean age at the onset of dementia was 15.8 ± 2.3 years (median 16, range 13–19). The earliest and main signs of cognitive deterioration were the negative effects on school performance, with a significant decline in academic achievement and mental slowing reported in all patients. Sometimes, the dementia was not accurately recognized or recognized early; specifically, Patients 1, 4, and 5a had been misdiagnosed with anxiety. After a mean of 6 months, cognitive impairment appeared more evident with the loss of the ability to conduct activities of daily living and the ability to perform tasks necessary for independent coping. Moreover, emotional (anxiety) and behavioral (irritability, aggressiveness, and hallucinations) disturbances were reported in all patients and were exacerbated by perampanel (up to 4 mg/day) in Patient 2, which was stopped within the first 5 months of treatment (22). In this phase, all patients showed different grades of mental deterioration (total intelligence quotient median 47, range 40–54), slow implementation, attention deficits, and significant impairment of executive function (in particular, patients 1, 3, and 4). Finally, cerebellar signs were also observed in this phase. The average elapsed time between the onset of cerebellar signs and epilepsy was 2.6 years (range: 1–5); the mean age at the onset of cerebellar signs was 15.6 ± 3.3 years (median: 17, range: 11–19). Both midline and hemispheric cerebellar signs were present, and a mild worsening of speech coincided with the onset of cerebellar ataxia. Falls were related either to ataxia or massive myoclonus. The mean disease stage was 2.6 ± 0.5 points (range: 2–3).

#### Phase 3: late stage

This late stage, reached by all LD patients except for Case 4, who is at the intermediate stage of the last follow-up, was characterized by a further worsening of the neurological picture, with a mean period of 7.2 ± 1.7 years (median: 6, range: 6–10 years) from the onset of epilepsy and a mean age of 19.6 ± 1.5 years (median: 19, range: 18–22). After 6 to 10 years from the epilepsy onset, pluri-monthly and drug-resistant myoclonic, tonic-clonic, and myoclonic-tonic-clonic seizures were associated with daily/pluri-daily myoclonic jerks (epileptic and non-epileptic). The mean myoclonus severity score was 4.8 (range: 4–5). Refractory status epilepticus (myoclonic, tonic-clonic, focal motor) was documented in cases 1, 2, 3, and 5b, and was sometimes responsive to phenytoin. Moreover, refractory status epilepticus, associated with pneumonia *ab ingestis* and percutaneous endoscopic gastrostomy (PEG)/tracheostomy placement, represented the transition from the intermediate to the late stage of the disease in all patients. Diffusely slow EEG superimposed on fast diffuse and multi-focal SW/PSW discharges, photosensitivity, and sequences of myoclonic jerks are often, but not always, associated with epileptiform abnormalities characterized by the EEG/polygraphic findings. All patients developed severe gait ataxia with incoordination and tremor associated with severe mental impairment (MOCA <10). The mean disease stage was 3.8 ± 0.4 points (range: 3–4).

#### Follow-up

The disease course was inevitably progressive. At the time of the last follow-up, Case 4 was at the intermediate stage, while the other cases were at the late stage. The mean follow-up duration after epilepsy onset was 6.5 ± 2.7 years (median: 6, range: 2–11) in all patients. Patient 1 was bed-bound with gastrostomy and tracheostomy, severe mental impairment, and complete dependence on others for activities of daily living, and the mean disease duration was 6 years. Patients 2 and 3 died of pneumonia, and the mean disease duration was 11 and 14 years, respectively; they were bed-bound with gastrostomy. Patient 5a died of probable sudden unexpected death in epilepsy (SUDEP); she was bed-bound with gastrostomy; the mean duration of symptoms was 11 years. Patient 5b died of sepsis associated with super-refractory status epilepticus; he showed severe mental impairment, severe gait ataxia, and complete dependence on other people for activities of daily living; the mean duration of symptoms was 6 years.

### Genetic findings

NGS analysis revealed the presence of mutations in the EPM2A gene in four families (families 1, 2, 3, and 4) and in the NHLRC1 gene in the remaining one (family 5). In detail, we identified two different point mutations in EPM2A (c.721C>T in families 1, 2, 3, and 4; c.243_246del in family 2) and only 1 in NHLRC1 (c.992del). An accurate description of each patient enrolled in the present study has been previously reported (3).

### MRI findings

Moderate and diffuse cerebral and cerebellar atrophy emerged after 3.3 years (range: 2–6) from disease onset; in Case 2, mild and diffuse cortical atrophy was already identified after 16 months after the disease onset.

### CSF findings

**Figure 2:**
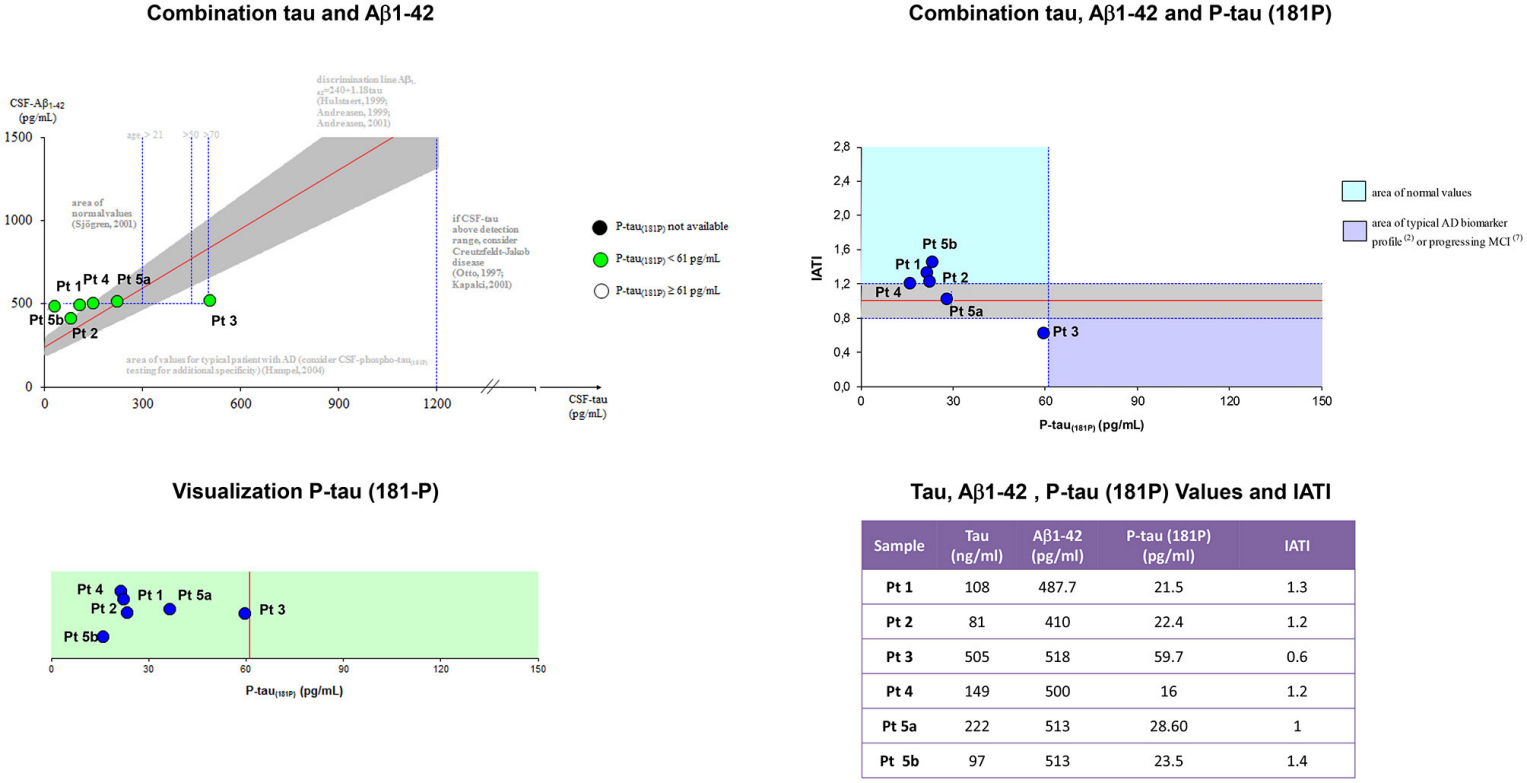
Aβ42, p-tau_181_, t-tauAg values, and IATI, and graphics of Aβ42, p-tau_181_, and t-tauAg visualization, and combination. According to the IATI index, the patients analyzed at the early stage showed a normal cognitive pattern profile (p-tau_181_ < 60 pg/ml and IATI > 1.2), while the patients at the intermediate and late stages showed a pattern of non-Alzheimer dementia (ptau_181_ < 60 pg/ml and IATI < 1.2).

Figure 2 showed Aβ42, p-tau_181_, t-tauAg values, and IATI, as well as graphics of Aβ42, p-tau_181_, t-tauAg visualization, and combination.

According to the IATI index, the patients analyzed at the early stage (patients 1, 4, and 5b) showed a normal cognitive pattern profile (p-tau_181_ < 60 pg/ml and IATI >1.2), while the patients at the intermediate (patient 3) and late stages (patients 2 and 5a) showed a pattern of non-Alzheimer dementia (ptau_181_ < 60 pg/ml and IATI < 1.2).

### ^18^f-FDG-PET findings

#### Interobserver agreement

All patients showed bilateral hypometabolic areas, with 100% agreement between the two readers. Overall, there was moderate agreement on region-based interpretation among the two readers (Cohen' κ 0.47) applying the graduated scale (preserved metabolism; mild, moderate, and severe hypometabolism). Most frequently, disagreement was observed between regions with mild and moderate hypometabolism.

#### Visual analysis

Per-patient analysis showed a trend for a difference between Phases 1 and 2–3 in PET metabolism, which was more prominent in Phases 2–3 (*p* = 0.051).

**Figure 3:**
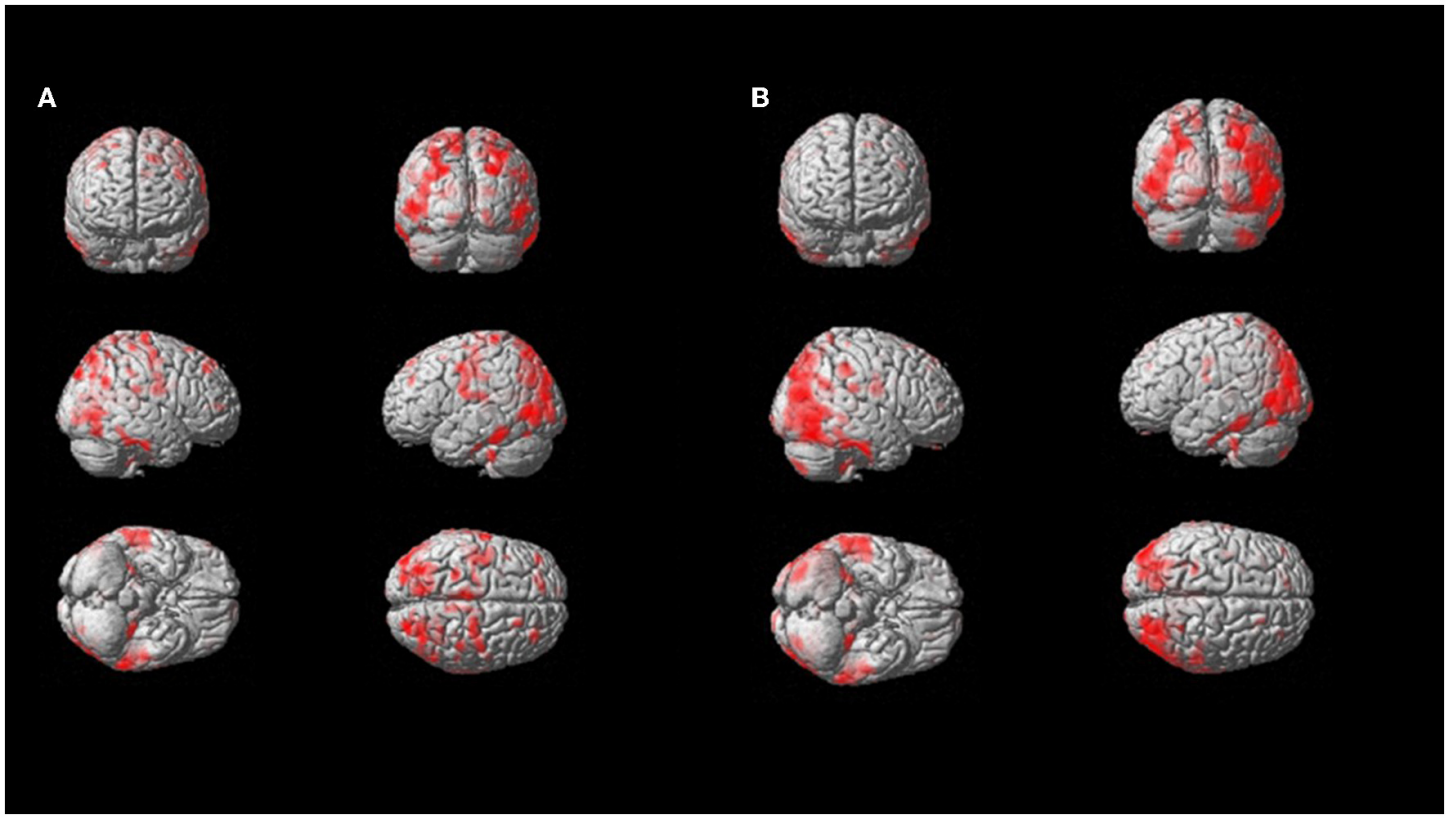
SPM patient 1 longitudinal analysis (*p* = 0.05 cluster-level above 100 voxels). SPM-t maps showed FDG hypometabolism in Phase 2 **(A)** and Phase 3 after 18 months **(B)**.

On a per-region analysis, hypometabolism was observed more frequently on the parietal lobe (10/11, 91%) and the posterior cingulate (10/11, 91%), the frontal lobe (9/11, 82%), and the praecuneus (9/11, 82%), and the thalamus (8/11, 73%) and the temporal lobe (8/11, 73%). A total of five out of six (83%) patients had another FDG-PET in a different phase of the disease. Among those, four out of five (80%) showed increased hypometabolism in the second FDG-PET compared to the first scan performed in a lower phase. The median number of regions positive for hypometabolism in Phase 1 was lower compared to the number of hypometabolic regions in Phase 2 or 3 (14 vs. 25 and 19, respectively). The Kruskal–Wallis test showed a statistically significant difference among the disease phases in PET results for the following areas: anterior cingulate, superior parietal, praecuneus, lateral temporal, mesial temporal, and thalamus (all *p* < 0.05; Figure 3).

**Figure 4:**
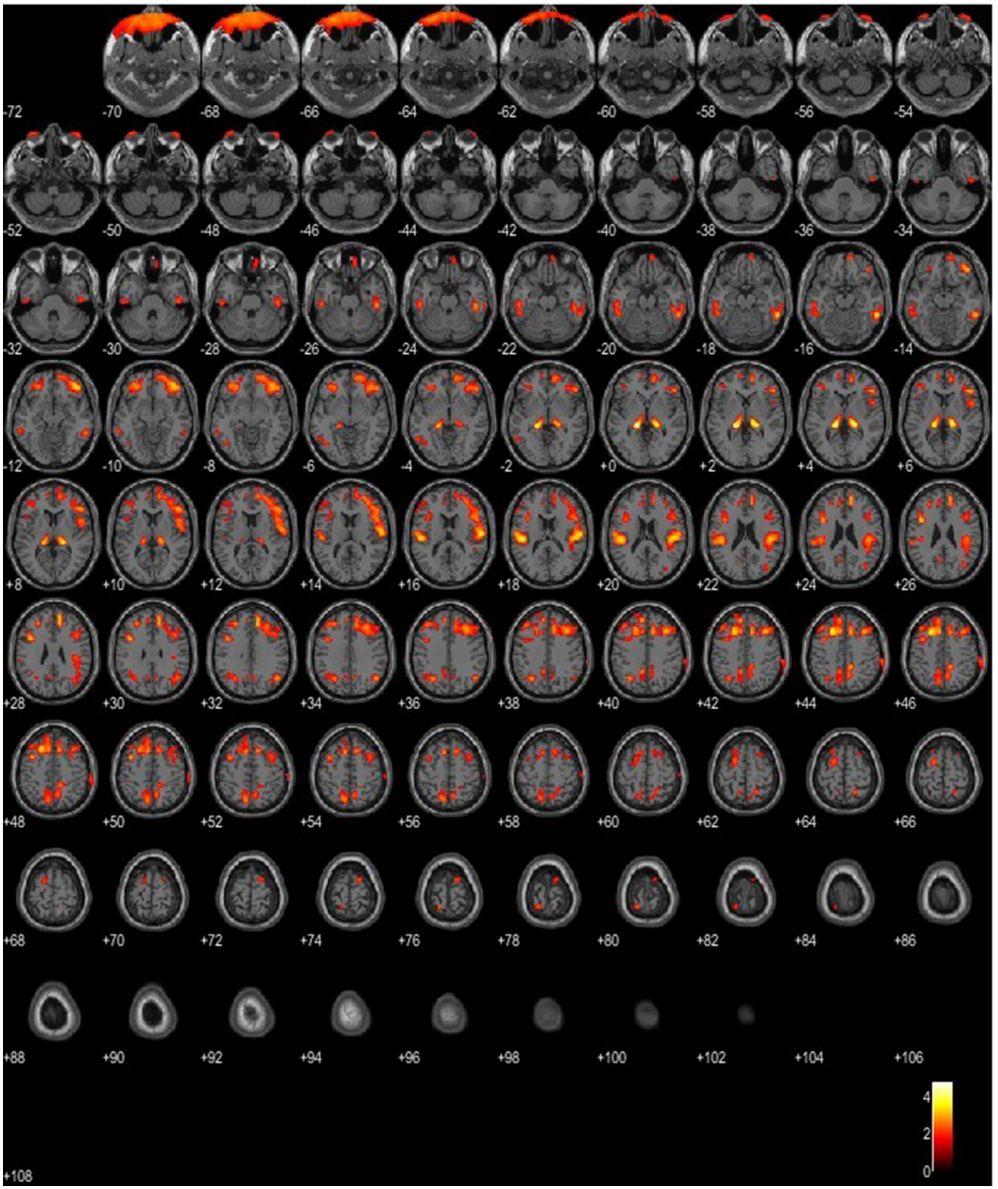
SPM patient 1 in Phase 2 of the disease (*p* = 0.05 cluster-level above 100 voxels). SPM-t maps show bilateral FDG hypometabolism within the thalamus and the temporal and frontal lobes.

On multivariable regression analysis, thalamus hypometabolism was associated with the presence of Phase 2 or 3 (*p* = 0.008) with a predictive model's efficiency of 90.9%. Similarly, lateral temporal lobe hypometabolism was associated with the presence of Phase 2 or 3 (*p* = 0.044; predictive model's efficiency was 81.8%; case example, patient 1, in Figure 4). Both of those regions showed a positive trend between the severity of hypometabolism and the phase of the disease.

#### SPM analysis

On SPM, the paired *t*-test, comparing patients with LD and the reference database, showed a bilateral hypometabolism within the thalamus. Interestingly, the paired *t*-test for hypermetabolism showed a bilateral increase in glucose metabolism within the putamen, which was more accentuated in patients harboring the EPM2A mutation.

### Amyloid PET

Overall, three ^18^F-flutemetamol PETs were performed. Both the amyloid PET scans conducted on patients in the most advanced stage (Phase 3) and that performed on one patient in the mild stage (Phase 2) did not reveal the presence of β-amyloid plaques.

## Discussion

The core findings are the following:

1. Three evolutive electroclinical stages, including four main progressive symptoms, have been outlined during the natural history of LD Apulian families.
2. Aβ (CSF Aβ42, amyloid PET) and neurodegenerative (CSF p-tau_181_ and t-tauAg, 18F-FDG PET) biomarkers indicate a pattern of cognitive impairment of the non-Alzheimer's disease type.
3. 18F-FDG PET is a promising biomarker to track progressive neurodegenerative changes occurring in LD and suggests the possible implication of the thalamus in LD pathogenetic mechanisms.

### Three electro-clinical stages in LD progression

Although progressive and fatal evolution of LD has been previously reported (1, 4, 5), full data on long-term electro-clinical follow-up are scarce, both for the rapid progressive evolution and delayed diagnosis.

After a mean follow-up duration of 6.5 ± 2.7 years, in our LD patients, we identified four main and progressive symptoms (epilepsy, myoclonus, ataxia, and dementia) included in three evolutive electroclinical stages.

The first stage is characterized by the onset of epilepsy, with tonic-clonic and focal visual seizures to bilateral tonic-clonic seizures, followed by myoclonic jerks, not exclusively upon awakening, associated with myoclonus, often postural and action-induced. The EEG background showed a slowing with superimposed diffuse epileptiform abnormalities, which are predominant in the occipital region. The second stage is characterized by a worsening of epilepsy and myoclonus associated with a gradual onset of dementia and cerebellar signs. All patients developed daily/pluri-daily myoclonic jerks, often associated with falls, and manifested drug-resistant epilepsy. The EEG background activity slowed further, with intermixed, diffuse, and faster discharges of SW/PSW during awakening and disorganized sleep; polygraphic recordings revealed action myoclonus and during posture maintenance and erratic myoclonic jerks at rest.

In the third stage of illness, which was observed in five out of six patients at a mean period of 7.2 ± 1.7 years from the onset of epilepsy, a further worsening of the neurological picture appeared, with pluri-monthly and drug-resistant myoclonic, tonic-clonic and myoclonic-tonic-clonic seizures, and refractory status epilepticus. All patients developed severe gait ataxia, became bed-bound, and developed severe dementia. Diffusely slow EEG, superimposed and fast diffuse and multi-focal SW/PSW discharges, photosensitivity, and sequences of myoclonic jerks are often, but not always, associated with epileptiform abnormalities characterized by the EEG/polygraphic findings. By the time of the last follow-up, three patients had died (due to sepsis, pneumonia, or SUDEP), and two were bed-bound.

### Non-Alzheimer's disease type dementia

Although cognitive deterioration is a key feature of LD, comprehensive studies on this type of dementia are very rare because the onset is subtle and the progression is rapid. However, all previous reports have described learning problems at school and a rapidly progressive ideo-motor slowing with severe impairment of executive functions, indicating the frontotemporal type of dementia (23, 24). Moreover, a predominant frontal cognitive impairment was entirely correlated with spectroscopic and PET findings (17, 24, 25).

In our cohort, dementia presented during the second electro-clinical stage, and the average elapsed time between dementia and epilepsy onset was 2.4 years. The earliest signs of cognitive deterioration were a significant decline in academic achievement, followed by mental slowing and the loss of the ability to conduct daily living activities and perform tasks necessary for independent coping. Concomitant emotions, especially anxiety, and behavioral disturbances (irritability, aggressiveness, and hallucinations) were also associated. The neuropsychological examination showed a prevalent executive impairment. Moreover, we also evaluated amyloid-β biomarkers (CSF Aβ42, amyloid PET) and 18F-FDG PET findings to further explore the type of dementia and to assess the role of these biomarkers in the progressive pathological process by evaluating them in the three electro-clinical stages.

According to the CSF findings and the IATI index, the patients analyzed at the early stage showed a normal level of CSF Aβ42 and a normal biomarker pattern profile (p-tau_181_ < 60 pg/ml and IATI >1.2), while the patients at the intermediate and late stages showed a normal level of CSF Aβ42 and a pattern of non-Alzheimer dementia (ptau_181_ < 60 pg/ml and IATI < 1.2), which is suggestive of a frontotemporal type of dementia. This is also partially supported by FDG-PET findings. Interestingly, the frontal and temporal lobes showed significant hypometabolism in the majority of patients, together with the parietal lobe, the posterior cingulate, the praecuneus, and the thalamus. These findings are in line with previous studies on FDG-PET in LD (17), confirming the dementia-like pattern of hypometabolism in our cohort of patients. Most importantly, SPM-based analysis supports the presence of bilateral thalamic hypometabolism as a key finding in those patients that warrants further studies to elucidate the physiopathology and the role of this sign. Moreover, ^18^F-Flutemetamol PET was consistent with CSF findings, as it did not disclose the presence of β-amyloid plaques in the early and intermediate phases. These original findings of amyloid-β biomarkers, correlating with neuropsychological testing, demonstrate a non-Alzheimer type of dementia in LD and apparently support the previous reports of a predominant frontal cortical metabolic involvement. Therefore, the combination of traditional CSF biomarkers (e.g., Aβ1–42/Aβ1–40 ratio, t-tau/Aβ1–42, and p-tau/Aβ1–42 ratios) may improve the diagnostic accuracy of cognitive decline in LD patients.

### PET as a biomarker of progression

A total of 80% of the LD patients showed more severe hypometabolism in the second FDG-PET compared to the first scan performed in a lower phase and more frequently on the parietal lobe and the posterior cingulate, the frontal lobe, the praecuneus, the thalamus, and the temporal lobe. Most importantly, the lateral temporal lobe and thalamus hypometabolism were associated with the presence of Phase 2 or 3.

Our PET findings support CSF and neuropsychological findings, indicating not only a non-AD type of dementia but also the implication of the thalamus in the pathogenetic mechanisms of LD.

Major limitations are the retrospective design and the low prevalence of the disease, which affects our cohort sample size and PET analysis robustness for disease progression. Furthermore, the frequent rapid progression of LD and its disabling evolution might be relevant for additional bias, namely the possibility of scanning only compliant and willing patients.

In conclusion, diagnosing LD as early as possible is essential because patients will be more likely to benefit from promising new therapeutic strategies if treated early in the disease course before major brain damage occurs. It is, therefore, important to develop biomarkers that are sensitive not only to this early stage but also to the progressive evolution of the disease. Our findings indicate that three electroclinical evolutive stages and FDG-PET are useful biomarkers for the onset and progression of LD. The combination of traditional CSF biomarkers improves the diagnostic accuracy of cognitive decline in LD patients, suggesting a cognitive impairment of the non-Alzheimer's disease type (frontotemporal type). Our findings need to be replicated in further studies to finely assess the temporal evolution of these associations and evaluate the efficacy of new promising disease-modifying treatments.

## Data availability statement

The original contributions presented in the study are included in the article/supplementary material, further inquiries can be directed to the corresponding author.

## Ethics statement

Ethical review and approval was not required for the study on human participants in accordance with the local legislation and institutional requirements. Written informed consent to participate in this study was provided by the participants' legal guardian/next of kin.

## Author contributions

Gd'O: study concept and design, analysis and interpretation, writing of the draft, and final revision. AF, LM, FB, OP, and MC: full text review, analysis and interpretation, and final revision. PP, SM, VA, VG, MD, ED, and MB: full text review and analysis and interpretation. All authors contributed to the article and approved the submitted version.

## References

[1] Turnbull J Tiberia E Striano P Genton P Carpenter S Ackerley CA. Lafora disease. Epileptic Disord. (2016) 18:S38–62. 10.1684/epd.2016.0842 27702709 PMC5777303

[2] Nitschke F Ahonen S Nitscke S Mitra S Minassian B. Lafora disease - from pathogenesis to treatment strategies. Nat Rev Neurol. (2018) 14:606–61. 10.1038/s41582-018-0057-0 30143794 PMC6317072

[3] d'Orsi G Lalla A Palumbo O Di Claudio MT Valenzano A Sabetta A. The presenting symptoms of Lafora disease: an electroclinical and genetic study in five Apulian (Southern Italy) families. Seizure. (2020) 83:145–53. 10.1016/j.seizure.2020.10.022 33152654

[4] Pondrelli F Muccioli L Licchetta L Mostacci B Zenesini C Tinuper P. Natural history of Lafora disease: a prognostic systematic review and individual participant data meta-analysis. Orphanet J Rare Dis. (2021) 16:362. 10.1186/s13023-021-01989-w 34399803 PMC8365996

[5] d'Orsi G Di Claudio MT Palumbo O and Carella M. Electro-clinical features and management of the late stage of Lafora disease. Front Neurol. (2022) 13:969297. 10.3389/fneur.2022.969297 36277909 PMC9580008

[6] Tassinari CA Bureau-Paillas M Dalla Bernardina B Picornell-Darder I Mouren MC Dravet C. La Maladie de Lafora. Rev EEG Neurophysiol. (1978) 1:107–22. 10.1016/S0370-4475(78)80126-9 96498

[7] Gómez-Abad C Gómez-Garre P Gutiérrez-Delicado E Saygi S. Lafora disease due to EPM2B mutations: a clinical and genetic study. Neurology. (2005) 64:982–6. 10.1212/01.WNL.0000154519.10805.F7 15781812

[8] Baykan B Striano P Gianotti S Bebek N Gennaro E Gurses C. Late-onset and slow-progressing Lafora disease in four siblings with EPM2B mutation. Epilepsia. (2005) 46:1695–7. 10.1111/j.1528-1167.2005.00272.x 16190947

[9] Singh S Sethi I Francheschetti S Riggio C Avanzini G Yamakawa K. Novel NHLRC1 mutations and genotype-phenotype correlations in patients with Lafora's progressive myoclonic epilepsy. J Med Genet. (2006) 43:e48. 10.1136/jmg.2005.039479 16950819 PMC2564581

[10] Singh S Ganesh S. Lafora progressive myoclonus epilepsy: a meta-analysis of reported mutations in the first decade following the discovery of the EPM2A and NHLRC1 genes. Hum Mutat. (2009) 30:715–23. 10.1002/humu.20954 19267391

[11] Riva A Orsini A Scala M Taramasso V Canafoglia L d'Orsi G. Italian cohort of Lafora disease: clinical features, disease evolution, and genotype-phenotype correlations. J Neurol Sci. (2021) 424:117409. 10.1016/j.jns.2021.117409 33773408 PMC8166462

[12] Franceschetti S Gambardella A Canafoglia L Striano P Lohi H Gennaro E. Clinical and genetic findings in 26 Italian patients with Lafora disease. Epilepsia. (2006) 47:640–3. 10.1111/j.1528-1167.2006.00479.x 16529633

[13] Bisulli F Muccioli L d'Orsi G Canafoglia L Freri E Licchetta L. Treatment with metformin in twelve patients with Lafora disease. Orphanet J Rare Dis. (2019) 14:149. 10.1186/s13023-019-1132-3 31227012 PMC6588886

[14] Turnbull J Epp JR Goldsmmith D Zhao X Pencea N Wang P. protein depletion rescues malin-deficient Lafora disease in mouse. Ann Neurol. (2014) 75:442–6. 10.1002/ana.24104 24419970

[15] Sanz P. Serratosa JM. Neuroinflammation and progressive myoclonus epilepsies: from basic science to therapeutic opportunities. Expert Rev Mol Med. (2020) 22:e4. 10.1017/erm.2020.5 32938505 PMC7520540

[16] Vincent A Macrì A Tumber A Koukas N Ahonen S Striano P. Ocular phenotype and electroretinogram abnormalities in Lafora disease. Neurology. (2018) 91:137–9. 10.1212/WNL.0000000000005821 29907606 PMC6059029

[17] Muccioli L Farolfi A Pondrelli F d'Orsi G Michelucci R Freri E. FDG-PET assessment and metabolic patterns in Lafora disease. Eur J Nucl Med Mol Imaging. (2019) 47:1576–84. 10.1007/s00259-019-04647-3 31858178

[18] Orsini A Ferrari D Riva A Santangelo A Macrì A Freri E. Ocular phenotype and electroretinogram abnormalities in Lafora disease and correlation with disease stage. J Neurol. (2022) 269:3597–604. 10.1007/s00415-022-10974-7 35184210 PMC9217906

[19] Magaudda A Gelisse P Genton P. Antimyoclonic effect of levetiracetam in 13 patients with Unverricht-Lundborg disease: clinical obervations. Epilepsia. (2004) 45:678–81. 10.1111/j.0013-9580.2004.56902.x 15144434

[20] Varrone A Asenbaum S Vander Borght T Booij J Nobili F Nagren K. EANM procedure guidelines for PET brain imaging using [18F]FDG, version 2. Eur J Nucl Med Mol Imaging. (2009) 36:2103–10. 10.1007/s00259-009-1264-0 19838705

[21] Friston KJ Passingham RE Nutt JG Heather JD Sawle GV Frackowiak RS. Localisation in PET images: direct fitting of the intercommissural (AC-PC) line. J Cereb Blood Flow Metab. (1989) 9:690–5. 10.1038/jcbfm.1989.97 2789231

[22] Canafoglia L Barbella G Ferlazzo E Striano P d'Orsi G Martino T. An Italian multicentre study of perampanel in progressive myoclonus epilepsies. Epilepsy Res. (2019) 156:106191. 10.1016/j.eplepsyres.2019.106191 31446282

[23] Cukiert A Vilela MM Scapolan HB Lefèvre BH Marques-Assis L. Mental deterioration in Lafora disease. Arq Neuropsiquiatr. (1990) 48:236–40. 10.1590/S0004-282X1990000200017 2124483

[24] Pichiecchio A Veggiotti P Cardinali S Longaretti F Poloni GU Uggetti C. Lafora disease: spectroscopy study correlated with neuropsychological findings. Eur J Paediatr Neurol. (2008) 12:342–7. 10.1016/j.ejpn.2007.09.008 18063398

[25] Villanueva V Alvarez-Linera J Gomez-Garre P Gutierrez J. MRI volumetry and proton MR spectroscopy of the brain in Lafora disease. Epilepsia. (2006) 47:788–92. 10.1111/j.1528-1167.2006.00526.x 16650146

